# Innovative Strategies of Training Mechanism of Higher Education for New Entrepreneurial Talents

**DOI:** 10.3389/fpsyg.2021.696978

**Published:** 2021-08-09

**Authors:** Fengyun Wei

**Affiliations:** Party School of CPC Shandong Provincial Committee, Jinan, China

**Keywords:** higher education, new entrepreneurial talents, training mechanism, innovative strategy, innovative training

## Abstract

With the development of the social economy, more and more talents are required in economic construction and innovation. The study aims to cultivate new entrepreneurial talents and promote the overall development of new ventures. First, the entrepreneurial ability of new entrepreneurial talents is analyzed, and the feasibility of improving the entrepreneurial ability of new entrepreneurial talents is verified. Second, the architecture is designed for improving the entrepreneurial ability of new entrepreneurial talents. About 350 employees in 20 ventures in Xi'an, Shaanxi Province are randomly selected as the subjects for a questionnaire survey (QS). Three-fifty questionnaires are distributed and 300 are collected. Descriptive statistics are used to analyze the 300 valid questionnaires. Then, the relationship between the psychological states of new entrepreneurial talents and their entrepreneurial ability is explored, and the influencing factors in the development of new entrepreneurial talents are analyzed. The results show that the number of employees in most ventures is between 250 and 400 and the age of the employees in the tested new ventures is between 35 and 40. More than one-third of employees are managers and over 86% of them have a degree of bachelor or above. The scores of the scale of the psychological state of new entrepreneurs are high. This shows that most of the new entrepreneurs have a high educational level, strong motivation for entrepreneurial success, and great self-confidence, which are essential in the process of innovative training. Therefore, the talent training of new entrepreneurial talents should be based on a high educational level and focused on practice.

## Introduction

Today, more and more individuals and groups are joining the entrepreneurial wave with the market economy reform of China, the rapid development of new technologies, and government incentives. The increasingly frequent entrepreneurial activities become the important driving force for promoting social and economic development (Li et al., 2018; Yi and Duval-Couetil, 2018). According to the Global Entrepreneurship Monitor (GEM), the early entrepreneurial activity index of China ranks first, and it is higher than that of developed countries, like the United States and Japan. It is also superior to that of emerging developed economies like South Korea. By the middle of the 21st century, newly established private enterprises and individual businesses in China will account for 90% of the total number of enterprises (Zhao and Lu, 2016).

New ventures are the source of national vitality. Key indices of an innovative country include initial innovation ability, knowledge innovation ability, and industrial upgrading ability. The cultivation of innovative talents can provide intellectual support for socioeconomic development, knowledge discovery, science and technology integration, and innovation guidance (Salter and Mckelvey, 2016; Bhagavatula et al., 2017). A report in the American Academy of Sciences (AAS) points out that the United States is facing unprecedented challenges in the development of its industry. The government cultivates their talents with leadership and entrepreneurial ability to be prospective industry pioneers to strengthen the national competitiveness of America (Sklaveniti, 2017; He et al., 2018). China also establishes a national innovative strategy, namely, the medium and long-term development outline, including the outline of the medium and long-term talent-cultivation plan, and the education reform and development plan (Zhang and Ma, 2018). The objective of the talent education of China is to cultivate high-quality professionals and top-notch innovative talents, and how to achieve the objective and excel in the new round of industrial technology revolution by cultivating the entrepreneurial ability of new entrepreneurial talents is the problem to be considered carefully (Valk, 2016; Richmond et al., 2017). Wu and Song (2019) argued that online training could save the cost of enterprises in the cultivation of entrepreneurial talents. With the advancement of Science and Technology (S&T) and the increasing demand for innovation, the scale of entrepreneurial talent cultivation is larger and larger, which leads to poor training quality. The entrepreneurial ability of many entrepreneurial talents does not improve after the training ends. The trend is still worsening. Therefore, the situation needs to be changed. With the advancement of S&T, global environmental change, and the demands of China for innovation and industrial upgrading, the cultivation of technological talents expands rapidly, while the cultivation of entrepreneurial talents is falling. Thus, the cultivation of technological and entrepreneurial talents poses a great challenge to entrepreneurship education in China.

Given the above, the architecture is designed for the cultivation of entrepreneurial ability of entrepreneurial talents by analyzing the relationship between psychological states, entrepreneurial ability, and the influencing factors in the process of improving the entrepreneurial ability of entrepreneurial talents. The study put forward the innovative modes for the cultivation of entrepreneurial talents, providing a basis for the rapid development of new ventures.

## Innovative Analysis on the Training Strategy of Entrepreneurial Talents

### Abilities of Entrepreneurs

Entrepreneurs should bear some basic abilities, namely self-regulatory ability, cooperation ability, innovative ability, and market analysis ability (Moldovan, 2017), as shown in Table 1.

**Table 1:** Abilities of entrepreneurs.

**Abilities**	**Attributes and contents**
Self-regulatory ability	It includes self-evaluation, monitoring ability, entrepreneurial intention, self-motivation, risk preference, commitment ability, implementation ability, professional ethics, and social responsibility.
Cooperation ability	The ability can attract high-quality talents, transfer entrepreneurs' views, and control and motivate talents under cooperation.
Innovative ability	This is an ability with forward-looking characteristics, with which products can be designed, improved, and manufactured.
Market analysis ability	It can identify business opportunities, propose strategies, provide analysis, marketing, and financial management, and can handle public relations.

**Figure 1:**
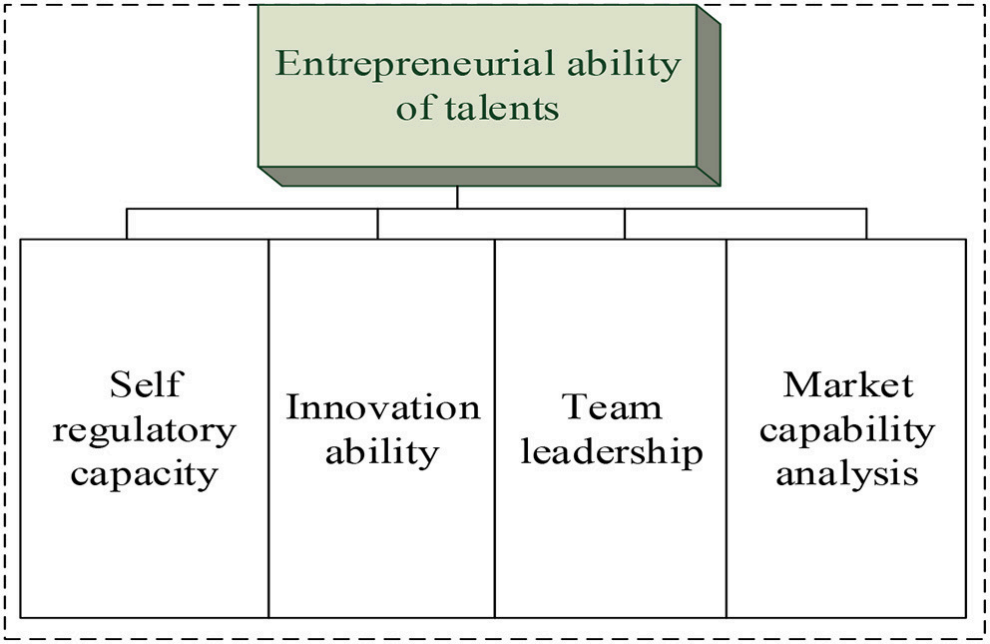
The model of the entrepreneurial ability of new entrepreneurial talents.

On this basis, the model of the entrepreneurial ability of new entrepreneurial talents is constructed, as shown in Figure 1.

### Feasibility of the Training Mechanism of New Entrepreneurial Talents

The feasibility of the training mechanism of new entrepreneurial talents should be evaluated, which is the basic work for strategic research. In this research, the feasibility of the training mechanism is evaluated by analyzing the relationship between entrepreneurial training and entrepreneurship education in colleges and universities.

In college entrepreneurship education, theories and practice should be combined to improve the abilities of students scientifically and technologically from the following two aspects. First, professional skills and technical management ability should be cultivated based on theories in various ways, including classroom teaching, lecture listening, and seminar participation, which are important in improving the entrepreneurial ability of entrepreneurial talents (Obschonka et al., 2020). Second, the teamwork ability of students should be cultivated by making them take part in team projects (Ghaferi and Dimick, 2016).

After that, it is found that more and more people with entrepreneurial potential have become entrepreneurs through education. Overall, entrepreneurs with good educational backgrounds are more likely to succeed in starting their businesses. Research results show that the students who take entrepreneurship courses tend to start a business earlier, and their enterprises have higher sustainability and growth rate (Boso et al., 2017). Some scholars believe that effective entrepreneurship education in colleges and universities enables students to identify and seize entrepreneurial opportunities better and succeed in managing business operations. Entrepreneurial ability can be cultivated through targeted training at colleges and universities (Hoppe, 2016; Hu and Wang, 2019). For college students with entrepreneurial potential, entrepreneurship education can help them build their knowledge system and improve entrepreneurial ability further, increasing the success rate of future entrepreneurship (Gast et al., 2017). This shows that the entrepreneurial ability of new entrepreneurs can be cultivated through education, that is, the entrepreneurial ability can be improved by entrepreneurship education.

### The Architecture of Cultivating the Entrepreneurial Ability of New Entrepreneurial Talents

First, the training plan for improving the entrepreneurial ability of new entrepreneurial talents is proposed. The goal is set in the entrepreneurial ability training, that is, the higher education plan for cultivating new entrepreneurial talents is made. Enough financial investment is provided to make the entrepreneurship education carry out smoothly in the colleges and universities. In addition, entrepreneurship courses are offered for college students, and entrepreneurship education is included in the basic education in colleges and universities.

Second, the mechanism of entrepreneurship education in colleges and universities is discussed, and it is analyzed from the dimensions of the education of frontier scientific knowledge, interactive teaching, entrepreneurial practice platforms, and effective feedback evaluation mechanism, as shown in Table 2.

**Table 2:** Mechanism of entrepreneurship education in colleges and universities.

**Dimensions**	**Main content**
Education of frontier scientific knowledge	It can encourage students to master cutting-edge scientific and technological knowledge, lead the development and operation of new products and new systems, and understand the importance and strategic impact of scientific research and technological development of society (Sarmiento, 2020).
Interactive teaching	It is guided by the interests and hobbies of entrepreneurial students. It solves the problems through autonomous learning and teachers' guidance and aims at overcoming difficulties and solving social problems.
Entrepreneurial practice platforms	Students can learn tacit knowledge through the practice platform to improve the practice ability of talents (Chen, 2018).
Effect feedback evaluation mechanism	A follow-up survey is conducted in the cultivation of new entrepreneurial talents, and the feedback is collected. The measures to evaluate the entrepreneurial ability in the cultivation of entrepreneurial talents are put forward, which is helpful to improving the training mechanism of new entrepreneurial talents (Krugh et al., 2020).

Third, the industry coordination mechanism is established. It can improve the entrepreneurial ability of new entrepreneurial talents through establishing industry-university cooperation strategic alliances and school-enterprise cooperation organizations, taking internship opportunities, and seeking financial assistance.

Fourth, the individual driving mechanism is constructed. It can reveal the psychological states (Zeng et al., 2020), the entrepreneurial motivation, and the willingness of new entrepreneurial talents (Ju and Zhou, 2020). Wu et al. (2019) showed that self-efficacy in entrepreneurial cultivation played a mediating role in forming narcissism, psychosis, and masculinism. The individual driving mechanism of new entrepreneurial talents is shown in Table 3.

**Table 3:** The individual driving mechanism of new entrepreneurial talents.

**Dimensions**	**Main content**
Psychological states	Positive psychological states can improve the entrepreneurial consciousness of new entrepreneurial talents and their entrepreneurial ability (Zhu et al., 2020). Entrepreneurial alertness and other psychological states affect their ability to identify entrepreneurial opportunities, which is mainly reflected in three aspects: the rational adventure, the moderate desire for achievement and, and the strong will. Qian et al. (2018) pointed out that employees' desire for achievement was positively correlated with their performance in doing a job, responsibility, and discourse power. Employees' states have a decisive impact on entrepreneurial success (Zhang et al., 2020).
Entrepreneurial motivation	It is the internal force to encourage and guide new entrepreneurial talents to take actions to achieve entrepreneurial success, carry out their entrepreneurial activities, and make entrepreneurial activities effective. To et al. (2020) revealed that competition could arouse entrepreneurs' entrepreneurial motivation and enhance their self-esteem. Chen (2019) suggested that entrepreneurial motivation could be stimulated under appropriate pressure, and improve their work performance. Strong entrepreneurial motivation helps to improve the entrepreneurial ability of new entrepreneurial talents.
Entrepreneurial intention	It is the tendency of new entrepreneurs' attitude toward entrepreneurial activities and the index to judge whether they will invest in entrepreneurial behavior. Also, it is the will, experience, and actions that guide new entrepreneurial talents to create business opportunities, determine their possibilities for entrepreneurial activities, and depend on individual power and social factors (Zaremohzzabieh et al., 2018).

### The Training Plan of the Entrepreneurial Ability of New Entrepreneurial Talents

(1) Training mechanism and research hypothesis of the entrepreneurial ability of new entrepreneurial talents.

**Figure 2:**
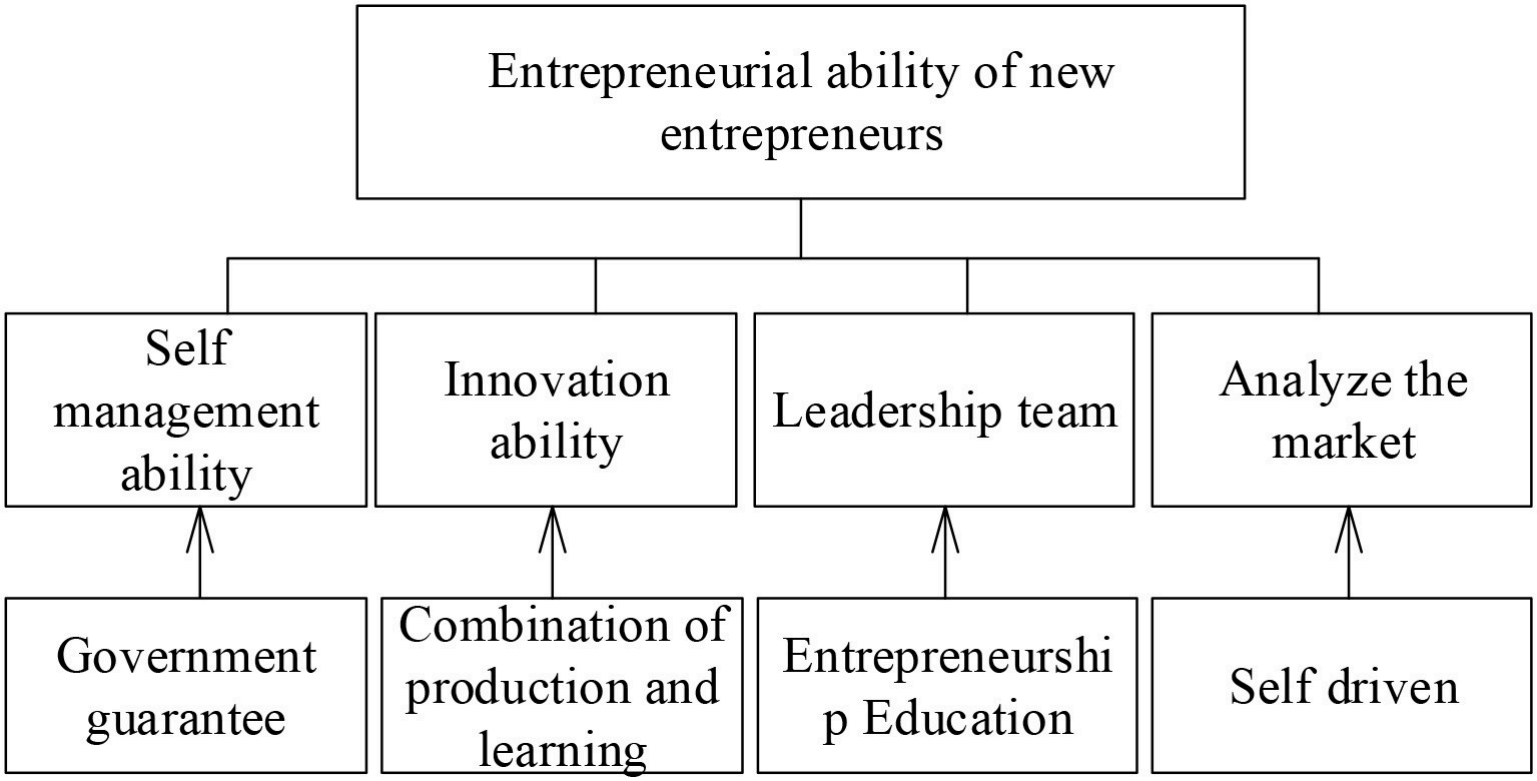
The initial model of training the entrepreneurial ability of new entrepreneurial talents.

According to the analysis of relevant influencing factors, the initial model of training the entrepreneurial ability of new entrepreneurial talents is established, as shown in Figure 2.

The following hypotheses are made for the relationship between the qualities of entrepreneurial ability and its influencing factors as shown in Table 4.

**Table 4:** Hypotheses on the relationship between the qualities and the influencing factors of the entrepreneurial ability of new entrepreneurial talents.

**Hypotheses**	**Content**
H1	There is a positive correlation between government security mechanisms and the personal ability of new entrepreneurial talents.
H2	There is a positive correlation between the mechanism of entrepreneurship education in colleges and universities and the personal ability of new entrepreneurial talents.
H3	There is a positive correlation between individual driving mechanisms and the personal ability of new entrepreneurial talents.

(2) The questionnaire survey (QS) is designed, distributed, collected, and processed. The questionnaire includes the basic information of the tested enterprises and the employees, the qualities of new entrepreneurial talents, and the influencing factors of training new entrepreneurial talents. At the same time, the psychological state scale of new entrepreneurial talents is designed, including the success motivation scale and the self-confidence scale. The contents of the success motivation scale and the self-confidence scale are shown in Tables 5, 6.

**Table 5:** The success motivation scale.

**Questions**	**Options**
Do you like doing novel and challenging tasks, even if risks are taken?	A. Never. B. Rarely. C. Not sure. D. Sometimes. E. Always.
Do you feel happy after finishing a challenging task?	A. Never. B. Rarely. C. Not sure. D. Sometimes. E. Always.
Are you attracted to do jobs that can show your intelligence?	A. Never. B. Rarely. C. Not sure. D. Sometimes. E. Always.
Do you prefer jobs that require your full potentials?	A. Never. B. Rarely. C. Not sure. D. Sometimes. E. Always.
Can you make your effort to uncertain tasks?	A. Never. B. Rarely. C. Not sure. D. Sometimes. E. Always.
Will you get deeply involved in a difficult task, even if it doesn't make sense?	A. Never. B. Rarely. C. Not sure. D. Sometimes. E. Always.
Are you feel challenged when you are faced with opportunities to show your ability?	A. Never. B. Rarely. C. Not sure. D. Sometimes. E. Always.
Are you attracted by difficult tasks?	A. Never. B. Rarely. C. Not sure. D. Sometimes. E. Always.
Are you attracted by uncertain jobs?	A. Never. B. Rarely. C. Not sure. D. Sometimes. E. Always.
Will you commence a task the moment you receive it, even if it costs much time?	A. Never. B. Rarely. C. Not sure. D. Sometimes. E. Always.
Are you attracted by the opportunities to show your ability?	A. Never. B. Rarely. C. Not sure. D. Sometimes. E. Always.
Do you feel excited and happy in front of uncertain tasks?	A. Never. B. Rarely. C. Not sure. D. Sometimes. E. Always.
Will you get interested in the tasks that you can't understand immediately?	A. Never. B. Rarely. C. Not sure. D. Sometimes. E. Always.
Do you prefer a challenging task, even if nobody does it?	A. Never. B. Rarely. C. Not sure. D. Sometimes. E. Always.
Do you like being assigned challenging jobs?	A. Never. B. Rarely. C. Not sure. D. Sometimes. E. Always.
Do you dislike working in uncertain situations?	A. Never. B. Rarely. C. Not sure. D. Sometimes. E. Always.
Do you worry about failures under uncertain circumstances?	A. Never. B. Rarely. C. Not sure. D. Sometimes. E. Always.
Do you worry about failures in doing a hard task?	A. Never. B. Rarely. C. Not sure. D. Sometimes. E. Always.
Do you feel upset when doing new and difficult jobs?	A. Never. B. Rarely. C. Not sure. D. Sometimes. E. Always.
Do you like the scenarios in which your ability is shown?	A. Never. B. Rarely. C. Not sure. D. Sometimes. E. Always.
Do you worry about your competency during doing uncertain jobs?	A. Never. B. Rarely. C. Not sure. D. Sometimes. E. Always.
Are you willing to do the things that you are unsure of?	A. Never. B. Rarely. C. Not sure. D. Sometimes. E. Always.
Do you feel uneasy when your ability is challenged?	A. Never. B. Rarely. C. Not sure. D. Sometimes. E. Always.
Do you worry about failures in the jobs that require you to seek opportunities?	A. Never. B. Rarely. C. Not sure. D. Sometimes. E. Always.
Do you worry about doing the jobs that look rather difficult?	A. Never. B. Rarely. C. Not sure. D. Sometimes. E. Always.
Are you reluctant to work in an unfamiliar environment?	A. Never. B. Rarely. C. Not sure. D. Sometimes. E. Always.
Do you prefer not to be assigned difficult tasks?	A. Never. B. Rarely. C. Not sure. D. Sometimes. E. Always.
Are you reluctant to do the jobs that exert all your potential?	A. Never. B. Rarely. C. Not sure. D. Sometimes. E. Always.
Are you reluctant to do the jobs that can show your competency?	A. Never. B. Rarely. C. Not sure. D. Sometimes. E. Always.
Do you get anxious when you encounter problems that can't be understood immediately?	A. Never. B. Rarely. C. Not sure. D. Sometimes. E. Always.

**Table 6:** Self-confidence scale.

**Questions**	**Options**
Do you think you are a valuable person, at least equal to others?	A. Never. B. Rarely. C. Sometimes. D. Always.
Do you think you have many advantages?	A. Never. B. Rarely. C. Sometimes. D. Always.
Do you think you are a failure in general?	A. Never. B. Rarely. C. Sometimes. D. Always.
Do you think you are as able as most people?	A. Never. B. Rarely. C. Sometimes. D. Always.
Do you think you are a proud person?	A. Never. B. Rarely. C. Sometimes. D. Always.
Do you think you have a positive attitude toward yourself?	A. Never. B. Rarely. C. Sometimes. D. Always.
Are you satisfied with yourself?	A. Never. B. Rarely. C. Sometimes. D. Always.
Do you respect yourself?	A. Never. B. Rarely. C. Sometimes. D. Always.
Do you feel you are useless sometimes?	A. Never. B. Rarely. C. Sometimes. D. Always.
Do you feel you are nothing sometimes?	A. Never. B. Rarely. C. Sometimes. D. Always.

The subjects are randomly selected and 20 new ventures in Xi'an Shaanxi Province are investigated. A total of 350 questionnaires are distributed, and 300 valid questionnaires are collected after the invalid are excluded. The recovery rate is 85.7%. After that, 30 enterprise managers are randomly selected to explore their psychological states.

(3) Descriptive statistics are used to test the reliability and the validity of the scale and conduct the variance analysis. The Statistical Product and Service Solutions (SPSS26.0) is applied to conducting descriptive statistics of the selected enterprises, including the establishment time, the number of employees, the types of jobs, and the age of the employees and their educational levels. Then, the reliability and validity of the questionnaire are analyzed, and variance analysis is conducted on whether there are differences in the entrepreneurial ability of new entrepreneurial talents and the influencing factors of cultivating new entrepreneurial talents. Origin 2018 64-bit is utilized to visualize the data.

## Results

### Reliability and Validity of the QS

**Figure 3:**
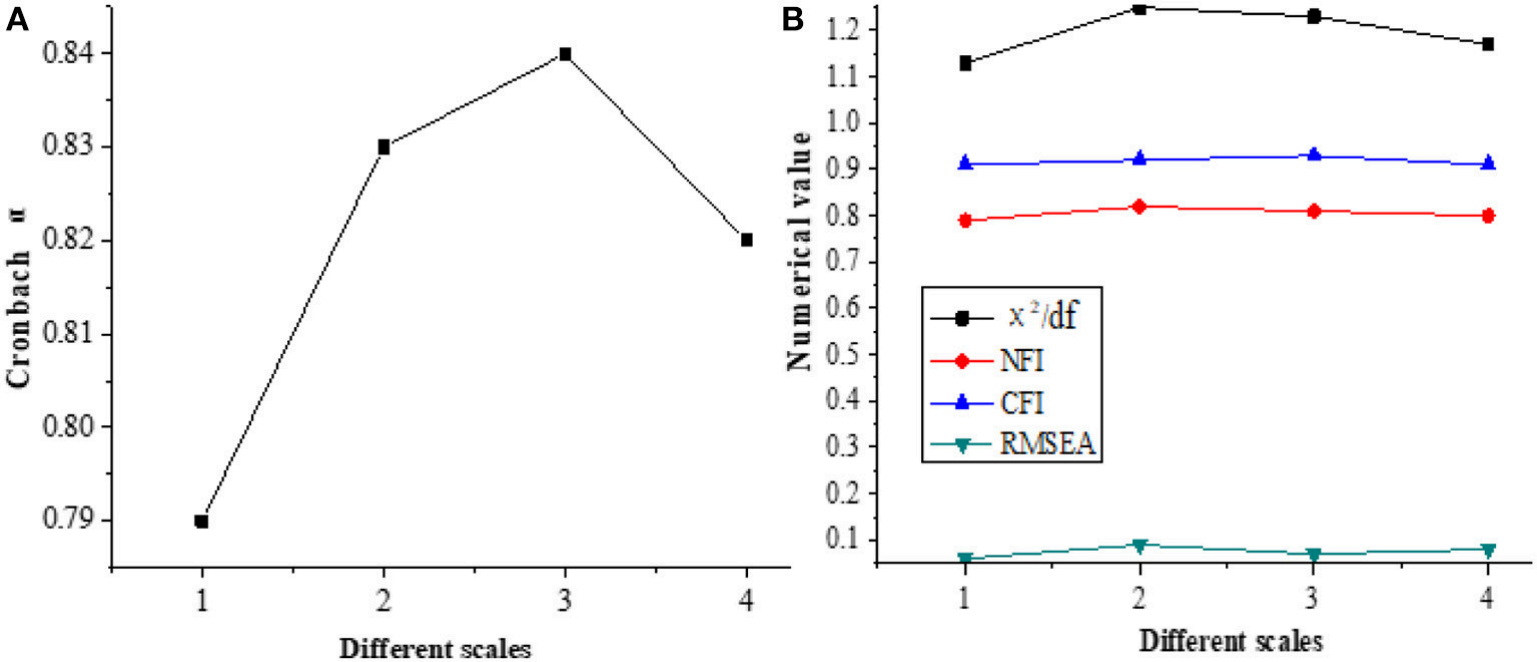
Reliability and validity of the questionnaire **(A)** Reliability; **(B)** Validity; 1. Scale of the qualities of the entrepreneurial ability of new entrepreneurial talents; 2. Scale of the influencing factors of cultivating the entrepreneurial ability of new entrepreneurial talents).

The reliability and validity of the questionnaire are tested, as shown in Figures 3A,B.

Therefore, the Cronbach α coefficient of each scale is between 0.79 and 0.85, indicating that the reliability of the scale is ideal. The χ^2^/df value of each scale is <2, and RMSEA is between 0.05 and 0.1, showing that the scale has a good fitting ability to the data and can be used in the study.

### The Establishment Time and the Number of Employees

**Figure 4:**
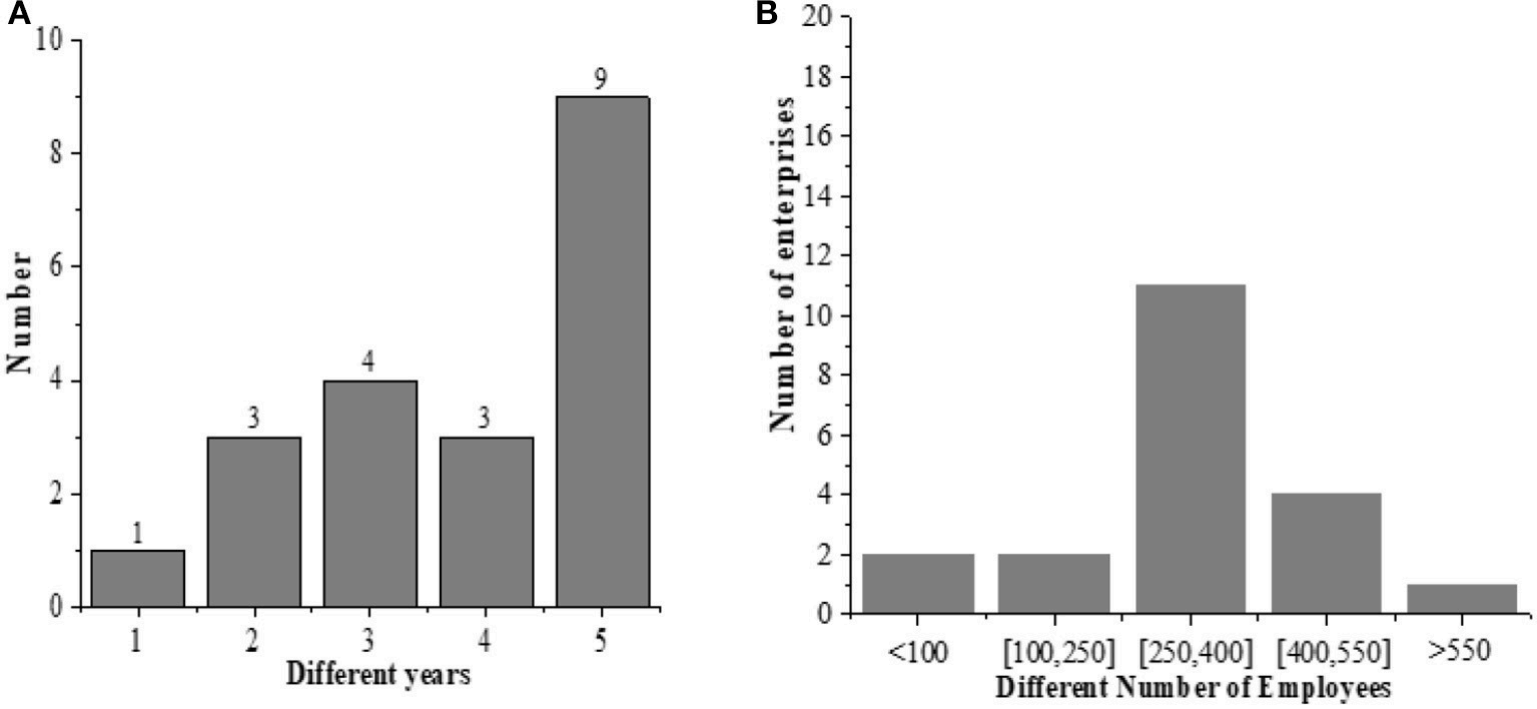
The establishment time and the number of the employees (**A**: the establishment time; **B**: the number of employees).

The establishment time and the number of the employees from the 20 enterprises are studied, as shown in Figures 4A,B.

The results show that the establishment time of one enterprise is 1 year, and that of three enterprises is 2 years, that of four enterprises is 3 years, that of three enterprises is 4 years, and that of nine enterprises is 5 years. The number of employees in two enterprises is <100, in two enterprises are between100 and 250, in 11 enterprises are between 250 and 400, in four enterprises are between 400 and 550, and in one enterprise are more than 550. This shows that the number of the employees in the surveyed new ventures is between 250 and 400, and most of the new ventures are established in about 5 years, which meets the requirements of new ventures.

### Nature of the Ventures and the Positions of Their Employees

**Figure 5:**
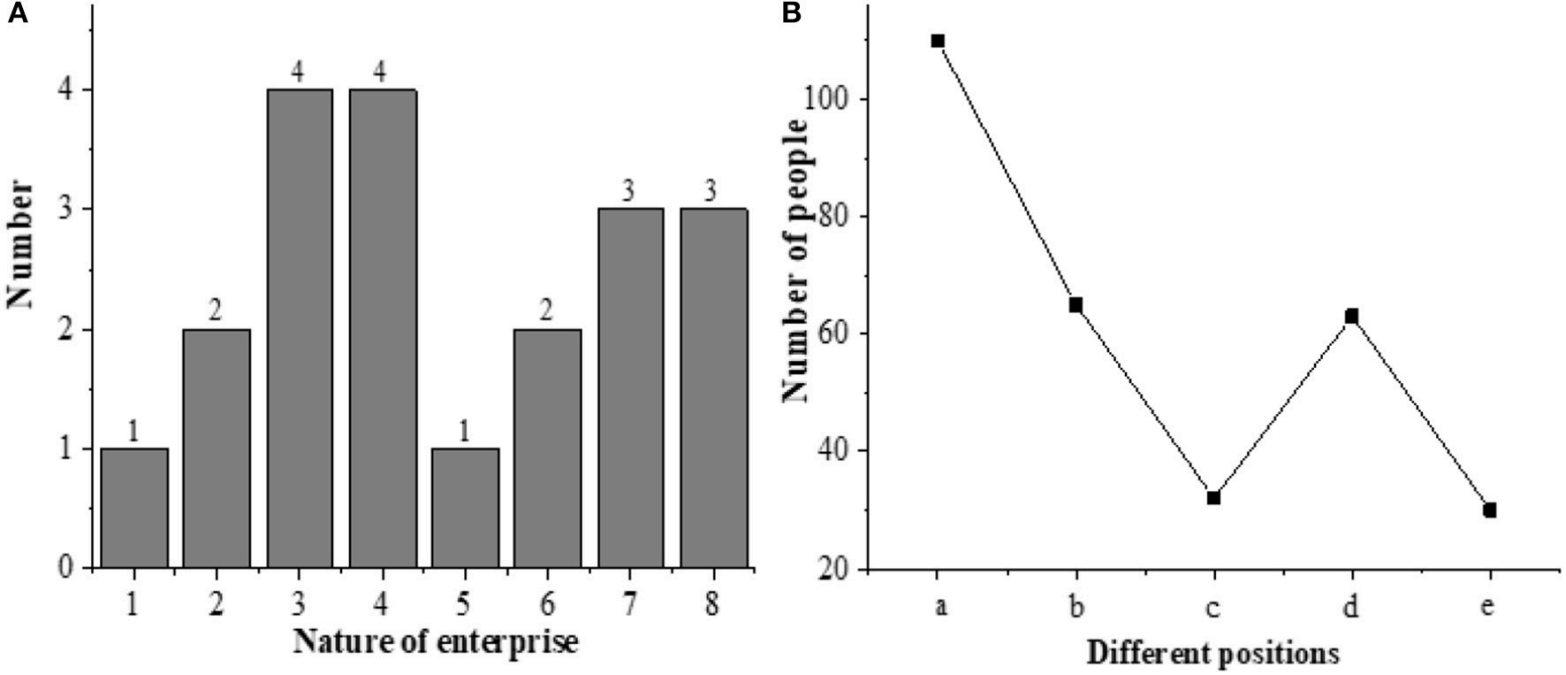
Nature of the new ventures and the positions of the interviewees (**A**. Nature; 1. A metallurgical company; 2. A construction company; 3. A software company; 4. A food company; 5. A biomedical company; 6. An energy and raw materials company; 7. A clothing company; 8. A water conservancy company; **B**. The positions of interviewees; a. Corporate executives; b. Salesmen; c. Production personnel; d. R and D personnel; e. After-sale employees).

The nature of the new ventures and the positions of the employees are statistically analyzed, as shown in Figures 5A,B.

Among the 20 enterprises, one is a metallurgical company, two are construction companies, four are software companies, four are food companies, and one is a biomedical company. Also, two are energy and raw material companies, three are clothing companies, and three are water conservancy companies. Among the 300 interviewees, 110 of them are corporate executives, 65 are sales, 32 are production employees, 63 are R and D personnel, and 30 are after-sale personnel. This shows that most of the surveyed new ventures are software companies or food companies, and most subjects in the ventures are managers, indicating that new entrepreneurial talents prefer the emerging industries, and they have strong working abilities.

### Age, Gender, and Education of the Subjects

**Figure 6:**
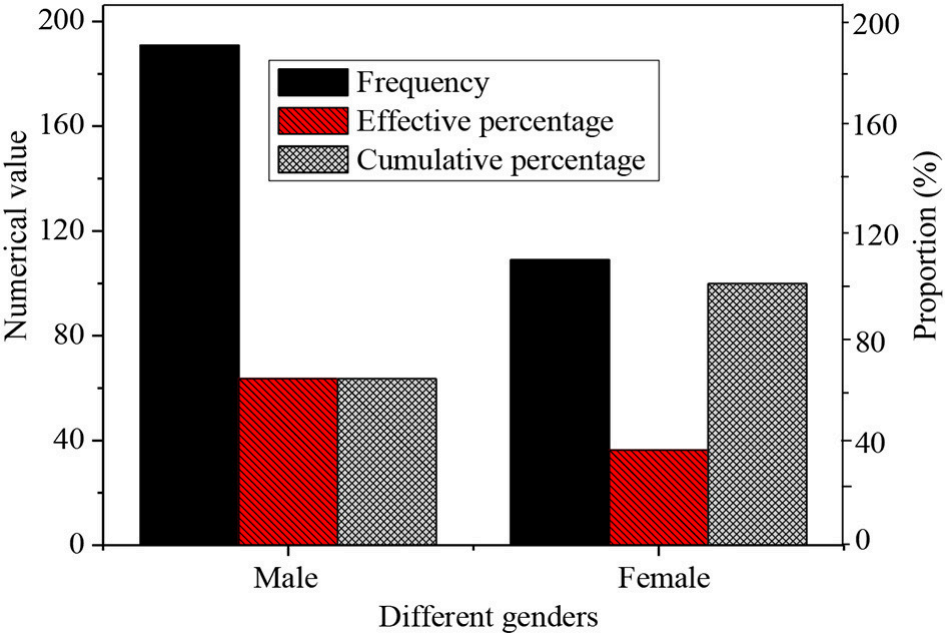
The genders of new entrepreneurial talents.

The genders of the 300 subjects are statistically analyzed, as shown in Figure 6.

Among the subjects, there are 191 male entrepreneurs and 109 female entrepreneurs, and the proportion of male-female is not balanced. Thus, different innovative strategies should be employed according to the gender ratio of new venture entrepreneurs. Effective policies should be provided to improve the entrepreneurial ability of female entrepreneurs, thereby improving the proportion of female entrepreneurs. In this case, H1 is verified.

The age and educational levels of the 300 employees are analyzed, as shown in Figure 6.

**Figure 7:**
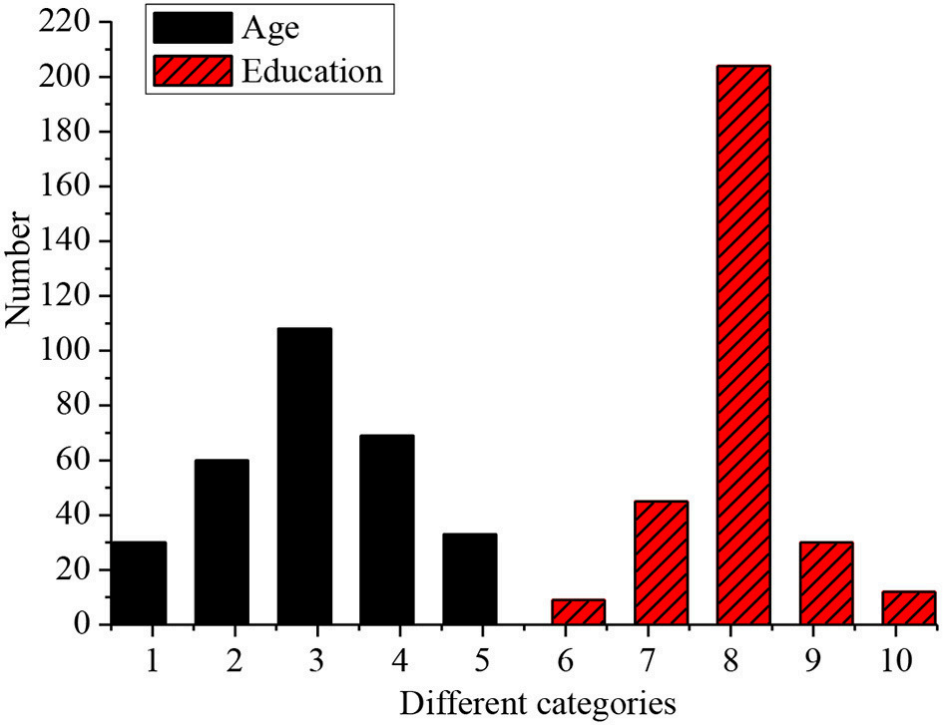
Age and education of the interviewees (1. <30 years old; 2. 30–35 years old; 3. 35–40 years old; 4. 40–45 years old; 5. Over 45 years old; 6. Below junior college degree; 7. Junior college; 8. Bachelor degree; 9. Master degree; 10. Doctor degree).

About 30 subjects are <30 years old, 60 are 30–35 years old, 108 are 35–40 years old, 69 are 40–45 years old, and 33 are over 45. There are nine subjects with a degree below a junior college, 45 with a junior college degree, 204 with a bachelor degree, 30 with a master degree, and 12 with a doctor degree. Most of the subjects are young people aged between 35–40 years, and most of them are managers. 82% of them have a bachelor degree or above. This indicates that the educational levels of new entrepreneurs are high as shown in Figure 7.

Therefore, young people with higher education should be cultivated and an entrepreneurial talent library should be established so that enterprises can use the strategies in the library to innovate their talent training mode. On this basis, H2 is verified.

### Psychological States of New Entrepreneurial Talents

**Figure 8:**
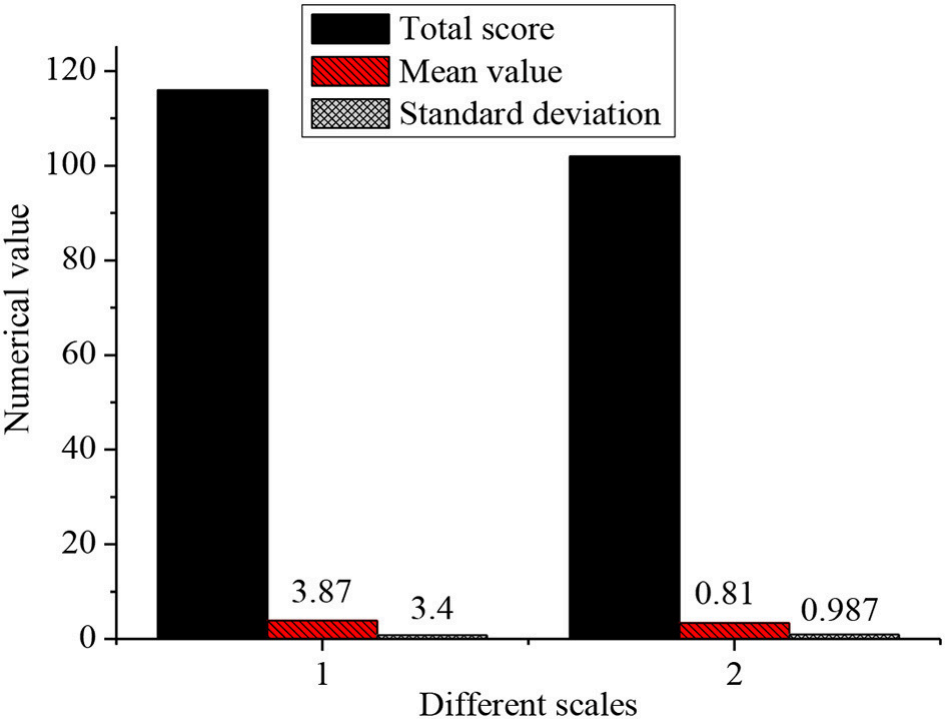
Psychological states of new entrepreneurial talents (1. Entrepreneurial motivation; 2. Self-confidence).

The psychological states of the subjects are statistically analyzed, as shown in Figure 8.

The total score of the entrepreneurial motivation scale is 116, with an average of 3.87 and an SD of 0.81. The total score of the self-confidence scale is 102, with an average of 3.4 and an SD of 0.987. The scores of both scales are high and H3 is verified. This indicates that new entrepreneurial talents have more self-confidence and strong entrepreneurial motivation during entrepreneurship, thereby greatly increasing the possibility of entrepreneurial success.

### Optimization of the Innovative Training Mechanism of New Entrepreneurial Talents

**Figure 9:**
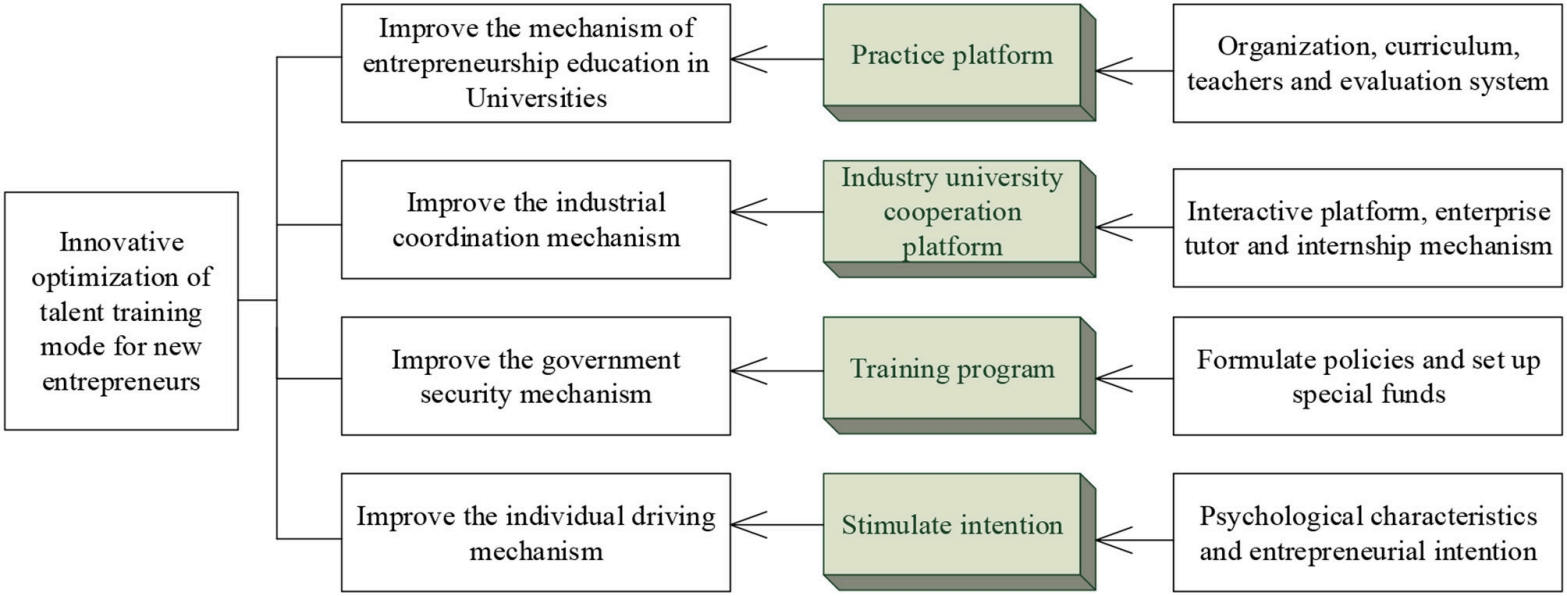
Optimization of the innovative training mechanism of new entrepreneurial talents.

The optimization of the innovative training mechanism of new entrepreneurial talents is carried out, as shown in Figure 9.

The training mechanism of industry-university cooperation should be optimized through the government security mechanism, with the entrepreneurial practice platform as the core and the strategic alliance of industry-university-research cooperation as the link. Meanwhile, the qualities and comprehensive ability of new venture entrepreneurs should be improved, and the training mechanism of industry-university cooperation is optimized. Besides, the individual driving mechanism should be optimized to arouse the entrepreneurial intention of new venture entrepreneurs. In short, based on the guarantees of the policies of governments, the entrepreneurship mechanism and the qualities of the talents play the greatest role in the innovative training of new entrepreneurial talents, and new entrepreneurial talents can achieve their optimal working ability and psychological capital.

## Conclusion

The training model is established to cultivate the entrepreneurial ability of new entrepreneurial talents, and some research hypotheses are proposed. The survey is conducted based on a QS. Then, the influencing factors of the psychological states of new entrepreneurial talents are analyzed. The results show that the number of employees in most of the surveyed enterprises is moderate, and more than a third of the subjects is the head of the enterprise. Meanwhile, most subjects in the new ventures are young people aged between 35 and 40, and the education that they receive is at least an undergraduate degree, and more than 80% of them have the education in colleges. The scores of psychological states of new entrepreneurial talents are good, indicating that in innovative training of new entrepreneurial talents, attention should be paid to improving the psychological states and practical ability of new entrepreneurial talents. There are some shortcomings in the study. The number of the research samples is small, which affects the applicability of the research results. In the later research, the sample size and scope will be expanded so that the research results will be more convincing and applicable.

## Data Availability Statement

The raw data supporting the conclusions of this article will be made available by the authors, without undue reservation.

## Ethics Statement

The studies involving human participants were reviewed and approved by Party School of CPC Shandong Provincial Committee Ethics Committee. The patients/participants provided their written informed consent to participate in this study. Written informed consent was obtained from the individual(s) for the publication of any potentially identifiable images or data included in this article.

## Author Contributions

The author confirms being the sole contributor of this work and has approved it for publication.

## Conflict of Interest

The author declares that the research was conducted in the absence of any commercial or financial relationships that could be construed as a potential conflict of interest.

## Publisher's Note

All claims expressed in this article are solely those of the authors and do not necessarily represent those of their affiliated organizations, or those of the publisher, the editors and the reviewers. Any product that may be evaluated in this article, or claim that may be made by its manufacturer, is not guaranteed or endorsed by the publisher.
